# DUVEL: an active-learning annotated biomedical corpus for the recognition of oligogenic combinations

**DOI:** 10.1093/database/baae039

**Published:** 2024-05-28

**Authors:** Charlotte Nachtegael, Jacopo De Stefani, Anthony Cnudde, Tom Lenaerts

**Affiliations:** Interuniversity Institute of Bioinformatics in Brussels, Université Libre de Bruxelles-Vrije Universiteit Brussel, Boulevard du Triomphe, CP 263, Brussels 1050, Belgium; Machine Learning Group, Université Libre de Bruxelles, Boulevard du Triomphe, CP 212, Brussels 1050, Belgium; Machine Learning Group, Université Libre de Bruxelles, Boulevard du Triomphe, CP 212, Brussels 1050, Belgium; Machine Learning Group, Université Libre de Bruxelles, Boulevard du Triomphe, CP 212, Brussels 1050, Belgium; Pharmacologie, Pharmacothérapie et Suivi Pharmaceutique, Université Libre de Bruxelles, Boulevard du Triomphe, CP 205, Brussels 1050, Belgium; Interuniversity Institute of Bioinformatics in Brussels, Université Libre de Bruxelles-Vrije Universiteit Brussel, Boulevard du Triomphe, CP 263, Brussels 1050, Belgium; Machine Learning Group, Université Libre de Bruxelles, Boulevard du Triomphe, CP 212, Brussels 1050, Belgium; Artificial Intelligence Laboratory, Vrije Universiteit Brussel, Pleinlaan 2, Brussels 1050, Belgium

## Abstract

While biomedical relation extraction (bioRE) datasets have been instrumental in the development of methods to support biocuration of single variants from texts, no datasets are currently available for the extraction of digenic or even oligogenic variant relations, despite the reports in literature that epistatic effects between combinations of variants in different loci (or genes) are important to understand disease etiologies. This work presents the creation of a unique dataset of oligogenic variant combinations, geared to train tools to help in the curation of scientific literature. To overcome the hurdles associated with the number of unlabelled instances and the cost of expertise, active learning (AL) was used to optimize the annotation, thus getting assistance in finding the most informative subset of samples to label. By pre-annotating 85 full-text articles containing the relevant relations from the Oligogenic Diseases Database (OLIDA) with PubTator, text fragments featuring potential digenic variant combinations, i.e. gene–variant–gene–variant, were extracted. The resulting fragments of texts were annotated with ALAMBIC, an AL-based annotation platform. The resulting dataset, called DUVEL, is used to fine-tune four state-of-the-art biomedical language models: BiomedBERT, BiomedBERT-large, BioLinkBERT and BioM-BERT. More than 500 000 text fragments were considered for annotation, finally resulting in a dataset with 8442 fragments, 794 of them being positive instances, covering 95% of the original annotated articles. When applied to gene–variant pair detection, BiomedBERT-large achieves the highest F1 score (0.84) after fine-tuning, demonstrating significant improvement compared to the non-fine-tuned model, underlining the relevance of the DUVEL dataset. This study shows how AL may play an important role in the creation of bioRE dataset relevant for biomedical curation applications. DUVEL provides a unique biomedical corpus focusing on 4-ary relations between two genes and two variants. It is made freely available for research on GitHub and Hugging Face.

**Database URL**: https://huggingface.co/datasets/cnachteg/duvel or https://doi.org/10.57967/hf/1571

## Introduction

Biomedical Natural Language Processing (bioNLP) is a field defined at the intersection of Artificial Intelligence (AI), Linguistics and Biomedicine that has been shown to be instrumental in the curation of biological resources by extracting pertinent information from texts to populate said resources (1). Biomedical Relation Extraction (bioRE), a subfield of bioNLP, is specifically important as it is used to identify and classify relations between biomedical entities in texts to allow one to quickly collect information relevant for different biomedical associations as, for instance, protein–protein (2, 3), drug–drug (4, 5), drug–protein (6, 7), chemical–disease (8), variant–disease (8, 9), gene–disease (10) or multiple types of biomedical interactions (10, 11). While this has led to many advances, progress towards higher-order relationship detection is hindered as there are only a few bioRE datasets (5, 7) that allow for the identification of n-ary relationships, i.e. involving more than two entities.

Here we aim to contribute to this next step by producing a dataset that considers gene–variant–gene–variant relationships, thus *n *= 4, that may be used to train models that allow for the identification of oligogenic relationships in text. While current datasets like BioRED (11) possess some variant–variant relations, they are however too few to build RE models, thus motivating the need for the current work. The recently curated Oligogenic Diseases Database (OLIDA) provides a means to this end as it contains a well-structured list of variant and gene combinations associated with genetic diseases, providing simultaneously the articles where this information was found (12). An oligogenic variant combination is here defined as a combination of at least two variants found in different genes which together cause a genetic disease. Its simplest form is the digenic variant combination, i.e. a combination of homozygous/heterozygous variants in two genes. Consequently, this original resource can be used to build an innovative bioRE dataset of oligogenic variant combinations that could in turn be used to create a system capable of identifying such combinations in new texts, providing essential assistance in the manual curation process that is used to build resources like OLIDA. In this work, we focus on the digenic variant combinations, as they are the easiest form of oligogenic variant combinations and most articles focus on such cases.

Yet, the development of high-quality datasets for bioRE represents a significant effort (4, 6, 11, 13). As in all statistical learning tasks, the quality of the datasets is a bottleneck for any downstream task, be it classification, entity recognition or relation extraction. The creation of an appropriate and relevant corpus for a bioRE problem requires knowledge in both (i) NLP, in order to clearly define the scope and format of the annotations, and (ii) biomedicine, to be able to correctly identify relations of interest. The first point forms a bottleneck due to textual assumptions where the relation needs to be found. For example, lots of datasets chose to restrict the region of interest to single sentences containing the potentially related entities. While this approach simplifies the annotation, it excludes various situations in which a relation appears across multiple sentences, which may be especially true for the bioRE task considered here. An additional issue is the time needed to annotate a corpus, given that nowadays deep learning techniques require a considerable amount of data to produce meaningful results (14).

In relation to the second point, the need for biomedical expert annotators with in-depth domain knowledge makes this process expensive, potentially discouraging the creation of new datasets (13). Clearly, any approach reducing this annotation cost will be useful for the creation of new resources. Active learning (AL) is providing the answer here (15). In AL, the model is trained on a small amount of labelled data and is then used to select in the unlabelled set of data what it considers as the most informative samples to be labelled. For example, the model may consider the instances which are difficult to predict as the most informative, or select the instances the most different from the currently labelled set in order to obtain a diverse labelled set. This cycle of training-selection-labelling is repeated until a desired performance or number of labelled samples is reached. First, the number of annotated samples may be considerably reduced as the samples selected for labelling would be more informative than if they were randomly selected. Second, the selection strategy ensures a higher quality in the resulting annotated set. A previous study explored the use of AL for bioRE with deep learning models and showcased the interest of using such methods, especially versus randomly selecting samples for annotation (16).

The end product of the AL-based labelling is the DUVEL (**D**etection of **U**nique **V**ariant **E**nsembles in **L**iterature) corpus. This corpus is annotated at the entity-level with genes and variants, and at the relation-level with the digenic association between them. Not only do we provide here the first corpus of bioRE describing digenic variant combinations involved in diseases, we also benchmark the DUVEL corpus on four state-of-the-art biomedical language models, providing insight in the performance gains obtained via fine-tuning. The results moreover will reveal that by using AL, a dataset can be produced that generates higher quality results than simply training on a random sample of the annotated data.

The contributions of this work are the following:

- We propose the first corpus of digenic variant combinations, publicly available for research.
- We showcase the results of an AL-augmented annotation framework for bioRE, which results in favouring the labels for the minority class compared to an annotation in random order, which obtained only 1% of positive samples compared to the 15% obtained with the AL selection strategy.
- We benchmark this dataset with several state-of-the-art models, including Bidirectional Encoder Representations from Transformers (BERT)-based models, demonstrating excellent performance for curation.

## Methods

### Building the corpus

The DUVEL corpus is built with the aim to train bioRE models to automatically detect gene and variant combinations responsible for oligogenic diseases in articles. As a starting point, we chose to annotate digenic variant combinations, i.e. relations involving two genes and at least two variants, in the articles linked to them. It must be noted that while we filtered the articles for those containing digenic variant combinations, it remains possible to also find oligogenic variant combinations involving more than two genes, which is the case for 19 of the articles considered here.

The OLIDA database gathers information about such variant combinations, including references where they can be found (12). Articles reported in OLIDA go through a rigorous manual curation protocol where information is manually extracted and integrated in the database after obtaining a consensus on the annotation by two curators, who have the necessary biomedical knowledge to make the assessment and score the quality of the reported combinations. In consequence, the time to obtain the information is long and complicated, motivating the support of an automatic relation extraction model for this biocuration task.

For the construction of the corpus, the version 2 of OLIDA, with articles published up to February 2022, was used. It consists of 318 research articles, containing 1229 oligogenic cases linked to 177 diseases, involving 2600 variants in 929 genes.

Precise information on the building process of the corpus is provided in the next sections.

#### Creating the fragments of texts

**Figure 1.**
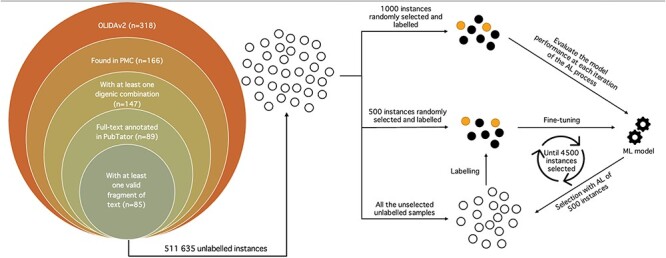
Schematic representation of the whole annotation process. Unlabelled instances are represented as unfilled circles, labelled instances as filled circles and the machine learning model as gears. The left picture depicts the filtering process of the OLIDAv2 articles, starting with 318 articles, to the 85 articles at the origin of the instances composing the DUVEL dataset. The right side represents the annotation with AL, with 1000 and 500 instances randomly selected from all the 511 635 unlabelled instances for the test set and initial labelled set, respectively. The labelled set is used to fine-tune the BiomedBERT model, whose performance is evaluated with the test set. The fine-tuned model selects 500 instances in the unlabelled instances, which it considers as the most informative according to the Margin selection strategy. The selected instances are then labelled and added to the labelled set for a new iteration of fine-tuning-selection-labelling. The process stops once 4500 instances have been selected and labelled.

OLIDA references (version 2.0) containing digenic combinations were retrieved. Biomedical entities of interest, i.e. genes and variants, were annotated for each full-text article with the help of the PubTator Central API (1). From the 318 research papers, only 166 possessed an entry in PubMed Central, essential for the use of PubTator. The papers were further filtered to keep those with digenic variant combinations, resulting in 147 papers. Only 89 of the 147 papers had their full-text annotated on PubTator and were ultimately used for the subsequent operations (Figure 1).

For each article, all possible combinations between two different genes and variants were generated. For each potential digenic variant combination, the sentences containing the entities involved in the combination were extracted, resulting in a fragment of the full text of the article, representing the shortest span of sentences possible. The fragment was excluded if its length, obtained with the tokenizer of Scispacy (17) which was trained and optimized for bioNLP, exceeded 256 tokens. Thus, a fragment of text can span from one to several sentences, as long as the number of tokens composing it is below 256. Only 85 papers possessed eligible fragments of text, resulting in a total of 511 635 fragments of text with a potential digenic variant relation (Figure 1).

As a pre-processing step, entity masking was performed, where each entity is masked with a type in the text, i.e. *@GENE$* and *@VARIANT$* for an entity belonging to the gene type and the variant type respectively, as was done previously in similar studies using BiomedBERT, previously named PubMedBERT (11, 18, 19). This ensures that the classification of the relationship relies on the context around the entities and not the entities themselves, producing a better generalization of the model (18, 20).

The resulting fragments of text are texts with masked entities, each containing a potential 4-ary digenic, i.e. gene–variant–gene–variant, relation.

Most of the articles contain a small number of variants and genes (less than one hundred), but the inherent nature of the digenic variant combination creates a combinatorial explosion of the number of candidates, as can be observed in Table 1.

**Table 1.** Statistics of the unlabelled corpus for the different entities and their combinations. The mean is calculated over the number of articles present in the corpus Numbers were rounded to the second decimal

Entities	Mean ± std	Total
Genes	12.79 ± 9.46	900
Variants	21.65 ± 20.63	1840
Gene combinations	69.93 ± 84.25	5693
(potential) Digenic variant combinations	6019.23 ± 15 537.51	511 635

Annotating all of these fragments would be costly in time and expertise. A comprehensive corpus would not need all instances, yet should provide a sufficient number of positive and negative instances so as to train a good classification model, thus motivating the use of AL.

#### Annotation with active learning

Annotations were done through ALAMBIC (21), an AL annotation interface that was run on a server with Ubuntu Desktop 20.04.5 LTS (GNU/Linux 5.15.0–56-generic x86_64) operating system, Nvidia driver 470.161.03, CUDA version 11.4, with 32GB RAM on 2 Asus GTX 1080 TI GPUs.

AL aims to annotate instances in the unlabelled set by selecting in an iterative way the most informative of those unlabelled samples. The AL process starts with an initial partially labelled set and is evaluated on a held-out test set. It then tries to infer the classes of the elements in the unlabelled set and extract the most interesting subset of unlabelled instances to be labelled. Once labelled, in our case by a human expert, the instances are added to the labelled set for a new round of training and testing. This cycle of training on the labelled set, testing on the held-out set, selection of the most informative instances and labelling of those selected instances is repeated until a specific number of samples are labelled (Figure 1).

The initial labelled set and held-out test set were created with respectively 500 and 1000 randomly selected instances. The AL process ends once the labelled set reaches a size of 5000 samples, meaning that 4500 candidates were selected through the AL strategy (Figure 1). The total annotation budget was thus 6000 samples, equivalent to the other n-ary bioRE datasets (5, 7). Standard precision, recall and F1-score metrics are used for evaluating the performance on the test set.

AL parameters and model are chosen according to the results of the experiments conducted on the fully annotated bioRE datasets (16) and additional experiments performed on the DDI dataset (4), a bioRE dataset with an imbalanced distribution as is also expected here (see Supplementary Method S1, Table S1 and Figure S1).

The model used during the AL process is the BiomedBERT model (18) coupled with a dense neural network with two outputs corresponding to the positive and negative classes. The BiomedBERT model is trained with a learning rate of 2e-5, for 3 epochs, and all the other hyperparameters following the original paper of Gu *et al*. (18).

Based on our previous experiments on deep learning in bioRE (16), the AL process is performed with the Margin selection strategy (22, 23) which performed the best on both balanced and imbalanced datasets in the experiments on established bioRE datasets (16). The Margin selection strategy selects the instances with the smallest difference between the predictions for the first and the second most likely class probabilities, i.e. the instances the closest to the decision boundary, meaning those where the decision was the most difficult to take.

The active batch size, i.e. the number of samples selected at each AL iteration round, is 500, a choice based on the results obtained on the DDI dataset (see Supplementary Method S1, Table S1 and Figure S1), in order to optimize the resulting performance, and minimize the computation time and number of iterations necessary to reach the total budget size.

#### Annotation process and guidelines

**Figure 2.**
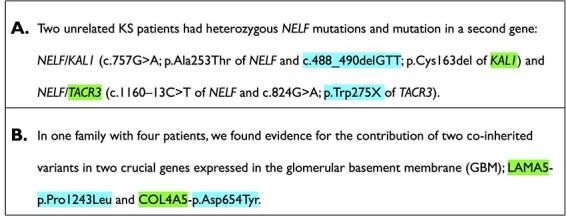
Examples of the different classes. Genes and variants are highlighted in the text. (A) Example of a text for the negative class. In this example, the variants and genes belong to two different patients and thus are not involved in a digenic variant relation. (B) Example of a text for the positive class. The text clearly states that the gene–variant pair is co-inherited in patients. The digenic variant combination depicted here is OLI504, more information can be found on https://olida.ibsquare.be/detail/Combination/OLI504/.

Annotation was conducted by a single annotator, one of the curators of the resource OLIDA (12), who had thus all prior biomolecular and medical expertise for the identification of digenic relationships in the text. The annotator had access to the genes and variants, the PMCID of the article the text was extracted from and the text with the masked entities. One out of three possible labels is assigned to each variant combination candidate:

- *0* for the absence of a digenic variant combination relation in the fragment of text.
- *1* for the presence of a digenic variant combination relation in the fragment of text. A relation exists only if the genes and the variants are relating to each other (see example in Figure 2). If the entities are involved in an alleged digenic relation according to OLIDA, but the syntactic aspects of the text showed no clear relation between the entities, then the fragment was deemed to not contain a relation. The combination needed to be carried by at least one individual.
*−1* if the fragment of text is not valid. The text can be deemed as invalid if one of the entities is not a valid entity, i.e. not a valid gene name or mutation, or the text contains an unfinished sentence or invalid sentence, i.e. with part of the text being a table. Invalid gene name and mutation comprised: (i) error in the annotation, e.g. P05, a patient denomination, which was annotated as a gene name or the cell line HEK293 which was annotated as variant; (ii) genes in species not human; (iii) Isoforms denominations of proteins and (iv) gene products. All excluded patterns can be found in the Supplementary Method S2. Tables were excluded as they are not considered as comprehensive text without the notion of their structure. To be used, they would need to be parsed in order to convey this structure.

Only instances from the positive and the negative classes (labels of 0 and 1) are included in the final dataset, all the invalid instances are excluded from further use as they are considered to not meet the quality standards.

It must be noted that it is possible to also find oligogenic variant combinations involving more than two genes and/or two variants, despite having filtered the articles for those containing digenic relations. In that case, we considered a subset of those variant combinations, i.e. two gene–variant pairs which are connected in the text and are part of the variant combination, as valid digenic variant combinations and classified them as class *1*. Examples of members of the positive and negative classes are depicted in Figure 2.

### Fine-tuning experiments

Fine-tuning is an approach of transfer learning where we adjust the internal structure of a pre-trained model based on the information acquired on a new dataset. We fine-tuned with our DUVEL dataset a number of biomedical language models, each coupled with a dense neural network with two outputs corresponding to the positive and negative classes.

In order to determine the best hyperparameters for the fine-tuning experiments, we conduct a hyperparameter grid search using the development set and training set on a server with Ubuntu Desktop 20.04.5 LTS (GNU/Linux 5.15.0–56-generic x86_64) operating system, Nvidia driver 470.161.03, CUDA version 11.4, with 32GB RAM on 4 Asus GTX 1080 TI GPUs.

The Hugging Face library (24) and W&B (https://www.wandb.com/) are used to perform and track the fine-tuning experiments. The following hyperparameter values were chosen for the grid search: number of epochs (3, 5, 10), learning rate (1e-5, 2e-5, 3e-5, 5e-5, 7e-5) and warmup ratio (0.1, 0.5), with a fixed batch size of 16 and no weight decay. The train set was shuffled with a fixed seed for reproducibility purposes across the experiments. Standard precision, recall and F1-score metrics are used for evaluation. The models with the hyperparameters obtaining the best F1-score on the development set are evaluated on the test set. The details on our chosen fine-tuning hyperparameters, model size and duration of the fine-tuning processes can be found in Table 2.

**Table 2.** Hyperparameters used for the fine-tuning experiments per model, with the model size (M = million parameters) and the time taken for the fine-tuning experiments

Model (size)	Epochs	Learning rate	Warmup ratio	Duration
BiomedBERT (109.5 M)	5	1e-5	0.5	13 min 45s
BiomedBERT-large (335.1 M)	3	1e-5	0.5	24 min 32s
BioLinkBERT (333.5 M)	3	1e-5	0.5	24 min 17s
BioM-BERT (334.6 M)	3	1e-5	0.5	24 min 46s

Four language models, known for their promising results in bioRE based on the BLURB benchmark (18), were selected: BiomedBERT-abstract-fulltext (18), BiomedBERT-large-abstract (18), BioLinkBERT-Large (25) and BioM-BERT-PubMed-PMC-Large (25, 26). The BLURB benchmark is an established benchmark for PubMed-based biomedical NLP applications, with a leaderboard tracking the results of the models developed by the community (18) (more information on https://microsoft.github.io/BLURB/).

BiomedBERT follows the classical BERT architecture, based on a transformer, and was pre-trained using purely biomedical texts from PubMed and PubMed Central (PMC), both abstracts and full-text articles. Its corresponding large version, BiomedBERT-large, is based on the large version of BERT and was pre-trained from scratch only with the abstracts from PubMed. BioLinkBERT also has a BERT architecture pre-trained with the same data as BiomedBERT, but it also incorporates the knowledge of the links (i.e. citations) between the documents during the pre-training. BioM-BERT is based on the ELECTRA implementation of the BERT architecture and was trained with PubMed abstracts and PMC articles with a vocabulary trained on general domain knowledge, such as Wikipedia.

## Results and discussion

### AL-based annotation process produces a corpus with a sufficient attention to the minority class

**Figure 3.**
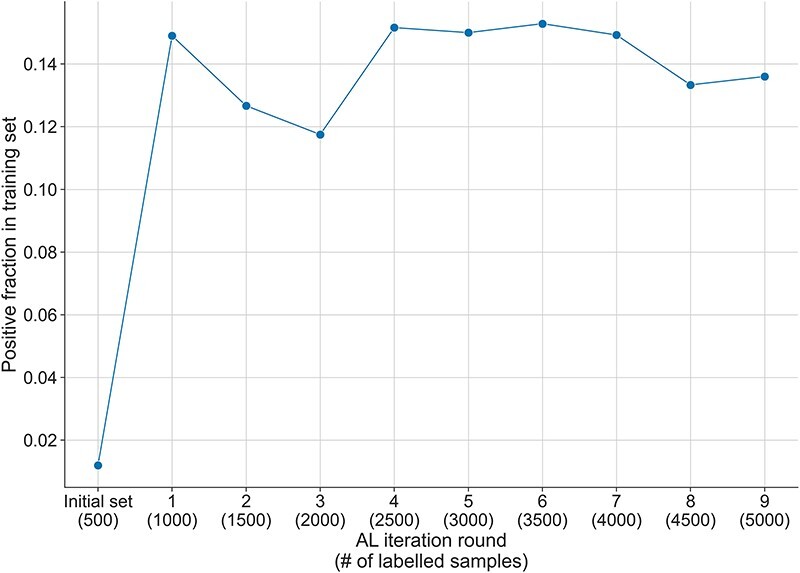
Fraction of positive samples in the training set across the AL iterations. The initial randomly selected labelled set has a positive fraction of 1%, which increases to more than 14% after the first round of AL selection and the addition of the 500 samples. The fraction of positive instances decreases slightly during the next two rounds of AL, but after that reaches and maintains a positive fraction between 13% and 15% until the end of the AL process.

During the annotation process, 314 390 samples were determined as invalid and were excluded from the pool of unlabelled data. A high number of those came from errors in the pre-annotation of the entities, mainly for the identification of genes. Exclusion of these errors, as well as the tables which were often found in the text, allowed us to greatly decrease the number of valid unlabelled instances. This underlines the gap still existing in the performance of automatic tools for Named Entity Recognition and the attention to pre-processing that has to be paid when it is a part of an annotation pipeline. The final pool of valid unlabelled instances is thus reduced to a set of 197 245 text fragments. The exclusion of the errors caused several restarts of the AL-based annotation process. In consequence, we annotated more than 6000 samples in the overall annotation process. However, the presented results linked to the AL process (Figure 3 and Supplementary Figures S2–S5) are based only on the selected 6000 instances (1500 randomly and 4500 with the AL strategy) during the AL process. We decided to include all the instances we annotated in the final dataset.

While the initial fraction of positive instances in the starting training set was of 1.2% (see ‘Methods’ section), the AL selection dramatically increased the positive fraction up to 15% over all AL iterations (see Figure 3). This AL effect was also observed in our previous experiments in bioRE (16). Reducing the imbalance in a dataset is particularly important, as a too excessive imbalance may have negative effects on the learned discrimination patterns, thus hindering the generalisation of the learned models (27). By using an AL selection strategy, we thus obtain a dataset of higher quality than what we would have obtained with just a random selection, as will be demonstrated later in the ‘Result’ section.

While the positive fraction increases, thanks to AL, the performance of the model used in the AL process to train on the labelled set did not vary much, hovering around 0.35 for the F1-score on the test set (see Supplementary Figures S2–S5). That performance is most likely due to the small fraction of positive instances in the test set (0.7%), making their detection much more difficult.

Table 3 shows the resulting statistics for the DUVEL corpus. A total of 8442 samples were annotated, with 7648 samples for the negative class and 794 of the positive class. Out of the 85 annotated papers with potential variant combinations, 81 papers were included in the final dataset, with 62 papers containing both negative and positive class instances. From the four papers not included in the dataset, two papers (PMCIDs: PMC8600266 and PMC3267856) had only invalid text fragments. The first had patient information only found in tables, making for invalid fragments of text. The second paper had only 28 valid fragments of text with the term *Mbp*, standing for mega base pair, recognized as a gene by PubTator. Moreover, the digenic variant combinations were only present in a table in the Supplementary material. The last two papers not found in DUVEL (PMCIDs: PMC4769270 and PMC6975284) are also mainly composed of invalid fragment of texts (99.3 and 64.2%, respectively, resulting in only 63 and 48 valid fragments of text), which could explain why they had a lower probability to be selected.

**Table 3.** Statistics of the DUVEL dataset splits. The training, development and test splits are the result of a stratified split of the whole dataset based on the labels, in order to maintain a similar class distribution across the splits (around 9.4% of positive class). The resulting dataset and splits are available through Hugging Face https://huggingface.co/datasets/cnachteg/duvel

	Train	Test	Dev	Total
Number of instances	6553	1689	200	8442
Number of positive instances	616	159	19	794
Number of articles	79	75	51	81
Number of articles with positive instances	61	51	12	64
Number of articles with negative instances	78	73	50	79

We estimated the Inter-annotator agreement (IAA) by computing Cohen’s kappa coefficient (28) with 1000 instances randomly selected in a stratified manner from the whole DUVEL dataset, which were annotated by a second annotator. The coefficient is 0.78, which corresponds to Cohen’s interpretation as substantial agreement. The differences in the annotation were mainly due to the difficulty of attributing a digenic relation in the sentence to an individual, or when several sentences were involved in the relationship.

**Figure 4.**
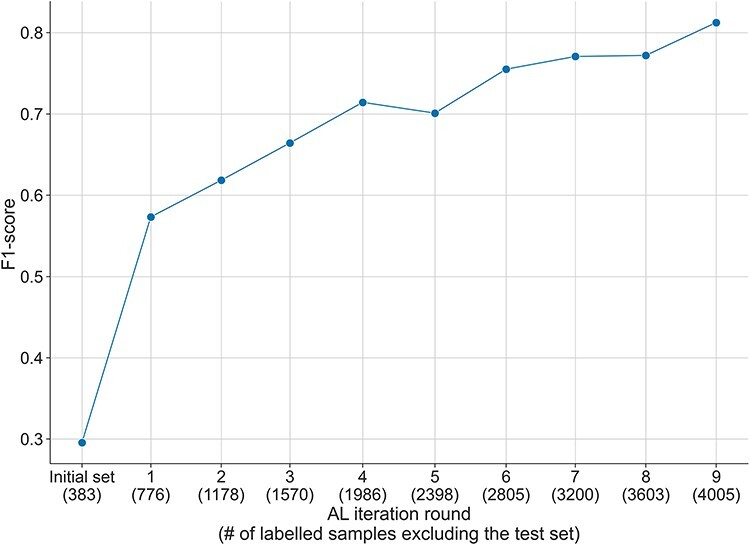
F1-score over the AL iterations during the annotation of the DUVEL data set, evaluated with the DUVEL test set instead of the test set initialised during the AL process. The initially selected samples during the AL process are filtered to exclude the samples present in the DUVEL test set.

In order to better evaluate the performance of the AL process, we measured the performance of the model during the AL process with the test set of the DUVEL corpus. We excluded from the selected instances at each round the instances in the DUVEL test set and used them to fine-tune the model from scratch. Contrary to the results obtained with the randomly selected initial test set used during the AL process (see Supplementary Figures S2–S5), in Figure 4, we observed an increase in the performance of the model from a F1-score of 0.3 at the initial labelled set to 0.8 with the final set of selected samples. Similar behaviors have been observed for the recall measure and in a lesser manner for the precision, which was less consistently improving over the AL iterations (see Supplementary Figures S6–S7). This proves the interest of using AL in order to obtain a high-quality dataset.

### Training SOTA RE models with DUVEL leads to high-quality performance

Table 4 displays the results of the benchmark experiments. Without fine-tuning, the models do not perform well, with BioLinkBERT having the worst performance, mainly due to its poor recall (0.006). Interestingly, BiomedBERT-large has a recall of 1 when not fine-tuned; however, has a really poor precision, indicating a tendency to label samples with the positive class indiscriminately.

**Table 4.** Performances of the models on the DUVEL test set Standard F1-score, precision and recall are used on the test set after fine-tuning or not of the models

Model	Fine-tuning	F1-score	Precision	Recall
BiomedBERT	No	0.1004	0.15	0.07547
	Yes	0.8171	0.7929	0.8428
BiomedBERT-large	No	0.1721	0.09414	1
	Yes	0.8371	0.8506	0.8239
BioLinkBERT	No	0.01047	0.03125	0.006289
	Yes	0.8207	0.7941	0.8491
BioM-BERT	No	0.08571	0.1765	0.0566
	Yes	0.8106	0.8592	0.7673

However, with the fine-tuning of the pretrained biomedical languages, all four models reach good performances, around 0.8 for their F1-score, similar to the performance attained at the end of the AL process. This demonstrates the necessity of a dataset for detecting such relations.

The highest F1-score is obtained with the BiomedBERT-large model (0.84), the highest precision at (0.86) with BioM-BERT, while BioLinkBERT had the highest recall (0.85). Despite its state-of-the-art results on the BLURB leaderboard for the bioRE, BioM-BERT had the lowest F1-score of the four models for DUVEL. The difference could be from the fact that while BioM-BERT also has a BERT architecture like BiomedBERT(-large) and BioLinkBERT, it was pretrained with the ELECTRA implementation of BERT (26). The ELECTRA implementation of BERT for pretraining uses a dynamic masking feature without using a next-sentence prediction objective, in the aim to train a better discriminator than the generator (29). This could be the reason why it has a higher precision than BiomedBERT and BioLinkBERT, as it could discriminate better the positive instances from the negative, at the cost of not identifying all the positive instances. The confusion matrices for the four fine-tuned models are available in the Supplementary Material (Supplementary Tables S2–S5).

In order to compare the performance of the dataset obtained with AL versus without AL (i.e. with Random Sampling), we performed fine-tuning experiments with five simulated datasets with a similar distribution as the initial training set and test set at the beginning of the AL process (around 1% of positive samples), assumed to follow the distribution of the whole unlabelled dataset (Supplementary Method S3 and Supplementary Table S6). Results showcased that even though the models did not exhibit null F1-score, recall and precision all around, the performances were rather poor for all the different metrics, generally with a higher precision than recall. The exception to those poor performances is the precision for BiomedBERT-large, which is very high (0.8), but is counterbalanced with a poor recall (0.09). This supports the hypothesis that the DUVEL dataset, built with AL is of a higher quality with its higher balance than what could have been obtained with Random Sampling.

### Exploring false positives and false negatives

The prediction errors were analysed for the fine-tuned BiomedBERT-large model, as it was the best performing model of the four in terms of F1. The typical error categories recorded are found in Table 5.

**Table 5.** Categories of errors found in the predictions of the DUVEL test set with the fine-tuned BiomedBERT-large model

Categories	Number of errors	Example
Missing context	19	Results molecular genetic testing of the ras/mapk pathway revealed a known pathologic heterozygous mutation in exon 12 of @ gene $ (@ variant $) and a novel missense variant in @ gene $ (@ variant $ ; Figure 3).
Negation not detected	3	The proband’s son (iii.1) has inherited the @gene$ @variant$ mutation, but not the @gene$/taci @variant$ mutation.
Complex sentence	21	Two unrelated ks patients had heterozygous nelf mutations and mutation in a second gene: nelf/kal1 (c. 757 g > a; p. ala253thr of nelf and c. 488 _ 490delgtt; p. cys163del of kal1) and nelf/tacr3 (@ variant $ of @ gene $ and c. 824 g > a; @ variant $ of @ gene $).
Same variant, different forms	2	He is a carrier of @ gene $ (mim 606 463 ; genbank: nm _ 001005741. 2 ; @ variant $) c. 1226a > g; @ variant $ and @ gene $ (mim 600 509 ; nm _ 000352. 4 ; rs151344623) c. 3989–9 g > a mutations.
Unknown	6	In patient avm359, one heterozygous vus (c. 589c > t [@ variant $]) in @ gene $ inherited from the mother and one likely pathogenic de novo heterozygous variant (c. 1592 g > a [@ variant $]) in @ gene $ were identified (Online Supplementary Table S2).

The BiomedBERT-large model made 51 errors on the 1689 instances of the test set, 23 false positives and 28 false negatives (see Supplementary Table S3 for the confusion matrix).

False positives are mainly found in texts where a valid variant combination exists, but the context fails to indicate that it is found in a patient, thus requiring a negative label. In this same category of errors, there also are combinations cited in an experimental setting, such as an *in vitro* experiment, making it difficult to know if the combination was positive or negative due to the missing patient context. Another case encountered is text where a negation affected part of the variant combination, which was not detected by the model. Negation is often difficult to handle for language models, and some strategies at the pretraining step need to be implemented to improve their understanding of negated sentences (30).

The false negatives are more often found in texts with multiple possible variant combinations or complex cross-sentence relations. Many studies have been working on the relation extraction task in cross-sentence texts, especially as they are generally rich in knowledge. Such problems are often tackled by transforming the text into a network based on its structural information, such as syntactic dependency, which already has had some promising results in bioRE, especially for n-ary relation (31, 32), and would be worthwhile to explore in the future with our dataset.

A small number of false negatives had an unknown origin, as the text is not particularly complex or is negated, and has all the context needed to determine if the digenic combination is valid and found in a patient.

## Conclusion

We present here the DUVEL corpus, the first biomedical corpus focusing on the 4-ary relation between two genes and two variants, thus more specifically on digenic variant combinations. We expect our corpus to serve as a new benchmark for n-ary cross-sentence bioRE, promoting research in bioNLP. It is made freely available for research on GitHub and Hugging Face.

We demonstrate the interest of using AL in a real-world setting, especially in an imbalanced scenario with a high number of unlabelled instances. With this process, we obtained a high-quality dataset and we demonstrated that good results can be obtained with different fine-tuned biomedical large language models. This will provide an important step forward in the curation of new publications for the resource OLIDA.

However, one needs to consider that the increase in balance obtained with AL comes with an increase in computation time to train the model after each annotation in order to select the next batch of instances to label. Moreover, using AL implies that there is a break between two batches of annotation, which could be inconvenient in a professional setting with paid annotators. Label Sleuth, an alternative AL-based annotation platform, tries to compensate for this problem by training in the background the model once a specific number of annotated sample is reached (33). Yet, next to more frequent updates of the model and the observation that the process that selects the instances needs to be adapted to such background updates, it also introduces an additional increase in computation cost that one should be aware of.

We limited the DUVEL corpus to the digenic case of oligogenic variant combinations, considered as the simplest, in order to work with a fixed length of four entities (i.e. two genes and two variants). However, oligogenic variant combinations are not limited to two variants and two genes involved in a relation, a clear limitation of our current work. Recently, Tiktinsky *et al*. (5) developed strategies to manage datasets with relations of variable n-ary lengths. Future work will explore how these strategies could be used to include variant combinations with a variable number of genes and variants.

Moreover, the maximum number of tokens in the fragments of text that contain the relation was limited to 256 in this work. Some bioRE datasets allow up to 512 tokens (4, 7, 8), and it can be argued that such a larger context can help the decision of the model, despite the fact that the relation can be contained in a single sentence (5, 31). While extending to a higher number of maximum tokens for fragments of text would dramatically increase the number of unlabelled instances, especially in our case with the inherent combinatorial aspect of oligogenic variant combinations, the use of AL in such context could help to select the more interesting subset of data to be labelled, and thus could also be a natural extension of our work. This interest will be explored further in the future.

To conclude, the DUVEL corpus provides an innovative resource that will speed up the development of new curation tools, which in turn produce data to generate progress in the many genetic studies of rare genetic diseases.

## Supplementary Material

baae039_Supp

## Data Availability

Datasets and code to reproduce the results in this article are freely available at https://github.com/cnachteg/DUVEL (doi:10.5281/zenodo.10987114). The dataset is also available on Hugging Face at https://huggingface.co/datasets/cnachteg/duvel (doi:10.57967/hf/1571). All results of the fine-tuning and error analysis are available on Zenodo (doi:10.5281/zenodo.10987237).
