# Supplementary material for: DUVEL: an active-learning annotated biomedical corpus for the recognition of oligogenic combinations
Source: Database (Oxford). 2024 May 28;2024:baae039. doi: 10.1093/database/baae039 (PMC11131422; doi:10.1093/database/baae039)
Supplement: baae039_Supp [file baae039_supp.zip › suppl_data/SupplementaryInformation.doc]

# Supplementary Information for

**DUVEL: An active-learning annotated biomedical corpus for the recognition of oligogenic combinations**

Charlotte Nachtegael, Jacopo De Stefani, Anthony Cnudde, Tom Lenaerts

**Corresponding author:**

Charlotte Nachtegael: Charlotte.Nachtegael@ulb.be

**This PDF file includes:**

Supplementary Methods S1 to S3

Supplementary Figures S1 to S7

Supplementary Tables S1 to S6

References for the Supplementary Information

# Supplementary Methods

## Supplementary Method S1: Experimental settings for the choice of active batch size

In order to find the optimal size for the active batch, i.e. the number of samples added at each iteration of the active learning process, for our active learning (AL) based annotation for the DUVEL data set, we simulated the AL process on the DDI data set [(1)](https://paperpile.com/c/PAVGdB/4hZc) with different active batch sizes. We follow the AL experimental specifications of [(2)](https://paperpile.com/c/PAVGdB/ICbO). The AL process was performed on the DDI data set with the original data splits. The initial labelled set was of size 100, taken from the training set. We used the Margin Selection Strategy as the AL [(3)](https://paperpile.com/c/PAVGdB/HadL) as the AL strategy and BiomedBERT [(4)](https://paperpile.com/c/PAVGdB/aqUV) as the base model, until a labelled set of size 5000 was reached.

The AL process was repeated 5 times with different initial labelled sets to obtain robust results. We tested 5 different active batch sizes: 10, 50, 100, 200 and 500. F1-score, accuracy, precision and recall were measured on the original test set after each training of the model with the labelled set. The plot of the average F1-score over the iterations can be found in Supplementary Figure S1. Performances of the different diverse active batch sizes are compared with the Area Under the Learning Curve [(5)](https://paperpile.com/c/PAVGdB/FiSO) and the average performance of the AL processes in function of the size of the labelled set in the Supplementary Table S1.

## Supplementary Method S2: Excluded patterns from the annotation

### Genes

- 'ALS'
- 'AIS'
- 'myc'
- 'tub'
- 'Gcn5'
- 'GnRH'
- 'connexin'
- 'NTD'
- 'P05'
- 'pLI'
- 'gata4'
- 'VEGF'
- 'S1, Table 1, and Figure 1'
- 'Amyotrophic Lateral Sclerosis'
- 'lemd3'
- 'MAF < 1%'
- 'MAF'
- 'enam'
- 'ambn'
- '2\.1'
- 'beta-catenin'
- 'Chr'
- 'GERP'
- 'helix'
- 'fog'
- 'USH2'
- 'P105'
- 'p200'
- 'p50 '
- 'insulin'
- 'scrib'
- 'vangl2'
- 'celsr1'
- 'HH1'
- 'BSP'
- 'BSP+'
- 'P15'
- 'S1"'
- 'MODY'
- 'TGF-beta '
- 'USH3'
- 'P1,P2'
- 'gonadotropin-releasing hormone'
- 'hCG'
- 'I-1, II-2'
- 'CD1-3'
- 'CD1'
- 'HED'
- 'I:1 and II:2'
- 'transferrin'
- 'GH'
- 'rp1l1'
- 'c2orf71'
- 'foxc1'
- 'ARS'
- 'degeneration'
- 'growth hormone'
- ‘Mbp’

### Invalid ends of sentences

- '-p.'
- 'and'
- ')p.'
- 'p.'
- '(p.'
- '/p.'
- ';p.'

### Invalid starts of sentences

- 'K897T'
- 'Ser509fs'
- 'I2547T)'
- 'A5V)'
- 'C1554G)'
- 'M170I)'
- 'S345F)'
- 'D91A)'
- 'V153I)'
- 'R1049Q)'
- 'K41I)'
- 'K238E)'
- 'P67A)'
- 'R485W)'
- 'P525L)'
- 'G287S)'
- 'T14I)'
- 'G1122S'
- 'Q493*'
- 'P340S* '
- 'G1108E'
- 'K618R	'
- 'Asn41Asp '
- 'R1057C '
- 'R769W '
- 'S323N) '
- 'Y1599*
- 'Q2924H'
- 'G644 V'
- 'R497Q*
- 'Val1259Ile
- 'G38R)'
- 'A1177G'
- 'Met210Ile)'
- 'C108Y,'
- 'A435T;'
- 'R148Q	'
- 'G323A ('
- 'Leu10Met '
- 'Pro133Alafs*13)'
- 'C108Y homozygous'
- 'C108Y is'
- 'C108Y and'
- 'R1865H'
- 'P136L)'
- 'R583H and'
- 'T1249I)'
- 'Gly194Cys)'
- 'S123T;'
- 'R408C)'
- 'Y211*]'
- 'R1044Q'
- 'Lys566Ter)'
- 'R583H, '
- 'Leu593Val '
- 'R3277C '
- 'N2167D '
- 'S2000C '
- 'De novo	M12'
- 'LP AR This study'
- 'LP AD This study'
- 'C D D'
- 'C D B'
- 'C NA NA'
- 'B,'
- 'N D D'
- 'N NA NA'
- 'Mother	M7'
- 'De novo	M17'
- 'Pol P'
- 'P AD'
- 'Mother	M21'
- 'C B B'
- '. . .'
- 'P AR'
- 'PD LP'
- 'Pol '
- 'De novo	M16'
- 'De novo	M 15'
- 'DC'
- 'De novo	M22'
- 'VUS AR'
- 'PD VUS'
- 'LP XL'
- 'PD P',
- '	Comparison'
- '	PTPN11’
- '	Sample'
- 'List of genes with homozygous nonsynonymous'
- 'Gene Nucleotide change'
- '	Genetics finding'
- '	Clinical and pathological'
- 'Patient ID Age'
- '	Identified ECHDC1 variants '
- 'Functional prediction and conservation scores'
- 'codon'
- 'Variant type Predicted',
- 'Sample Genes with two pathogenic mutations'
- 'Gene Nucleotide'
- 'Non-carriers: '
- '	Identified @GENE$ variants '
- '	Amino acids physical'
- 'Testicular volume <5'
- 'Individual sex Age'
- '	TESCO'
- '	Chromosome',
- 'DNAH6 c.T'
- '@GENE$ c.T'
- 'DNAH6 @VARIANT'
- 'DNAH6 c.C'
- '@GENE$ c.C1820'
- 'DNAH6 c.G'
- '@GENE$ c.G'
- '@GENE$ @VARIANT$ p.'
- '0.00078'
- '	6 FLNA'
- 'rs12'
- 'rs803'
- '	8 MYO7A'
- '>C'
- '>A'

## Supplementary Methods S3: Comparison of performances obtained with Random Sampling and Margin Sampling

While the resulting DUVEL data set was obtained through the selection of the most interesting instances by the model in the AL process, in our case with the Margin Sampling selection strategy, one may wish to know what the results are without AL selection strategy, i.e., with Random Sampling.

As we obtained the initial training set and test set with random selection, we can assume that the distribution of those sets follows the true distribution of the whole unlabelled data set, thus around 1% of positive samples.

Based on this assumption, we can simulate a DUVEL data set obtained with Random Sampling by under-sampling the positive instances (from the 616 positive instances) of the original training set of DUVEL and over sample the negative instances (from the 5937 instances), in order to obtain a data set of 65 positive instances and 6488 negative instances (with 551 oversampled negative instances), resulting in a training set of 6553 instances with around 1% of positive samples.

We created 5 simulated data sets with this process and used them as training sets for the fine-tuning experiments with the same hyperparameters as described in Table 2 in the main manuscript. We report the average and standard deviations of the standard F1-score, precision and recall on the original DUVEL test set over the 5 simulated data sets in the

Supplementary Table S6.

# Supplementary Figures

**Supplementary Figure S1.** Average F1-score for AL processes over the repeats for each active batch size for the DDI data set during an active learning process conducted with an initial labelled set of size 100 up to 5000 with the Margin Selection strategy with an active batch size of 10, 50, 100, 200 or 500.

**Supplementary Figure S2.** Accuracy over the active learning iterations during the annotation of the DUVEL data set. The accuracy has the tendency to be high as the test set is mainly composed of negative samples.

**Supplementary Figure S3.** F1-score over the active learning iterations during the annotation of the DUVEL data set. The drop of F1-score at step 7 is due to the small number of positive samples in the test set (7 samples) which were all predicted as negative at that iteration.

**Supplementary Figure S4.** Precision over the active learning iterations during the annotation of the DUVEL data set. The drop of precision at step 7 is due to the small number of positive samples in the test set (7 samples) which were all predicted as negative at that iteration.

**Supplementary Figure S5.** Recall over the active learning iterations during the annotation of the DUVEL data set. The drop of recall at step 7 is due to the small number of positive samples in the test set (7 samples) which were all predicted as negative at that iteration.

**Supplementary Figure S6.** Precision over the active learning iterations during the annotation of the DUVEL data set, evaluated with the DUVEL test set instead of the test set initialised during the AL process. The initially selected samples during the AL process are filtered to exclude the samples present in the DUVEL test set.

**Supplementary Figure S7.** Recall over the active learning iterations during the annotation of the DUVEL data set, evaluated with the DUVEL test set instead of the test set initialised during the AL process. The initially selected samples during the AL process are filtered to exclude the samples present in the DUVEL test set.

# Supplementary Tables

| **Batch size** | **F1-score** | | **Accuracy** | | **Precision** | | **Recall** | |
| --- | --- | --- | --- | --- | --- | --- | --- | --- |
|  | **Mean** | **SD** | **Mean** | **SD** | **Mean** | **SD** | **Mean** | **SD** |
| **10** | 1609.6 | 126.2 | 3789.1 | 9.3 | **2480.5** | 41.0 | 1356.6 | 165.5 |
| **50** | 1771.4 | 255.6 | 3788.5 | 14.5 | 2469.9 | 106.3 | **1552.2** | 353.6 |
| **100** | 1744.3 | 220.7 | 3792.3 | 18.7 | 2440.3 | 175.7 | 1489.6 | 238.7 |
| **200** | 1646.9 | 198.1 | **3878.6** | 16.8 | 2417.4 | 159.0 | 1386.1 | 181.3 |
| **500** | **1784.7** | 120.8 | 3799.0 | 10.5 | 2306.7 | 178.0 | 1541.3 | 100.4 |

**Supplementary Table S1.** Average AULC and standard deviation over the repeats for each active batch size for the DDI data set during an active learning process conducted with an initial labelled set of size 100 up to 5000 with the Margin Selection strategy with an active batch size of 10, 50, 100, 200 or 500. Best AULCs per metric are in bold. SD stands for Standard Deviation.

|  |  | **Actual class** | |  |
| --- | --- | --- | --- | --- |
|  |  | Positive | Negative |  |
| **Predicted class** | Positive | 134 | 35 | 169 |
|  | Negative | 25 | 1495 | 1520 |
|  |  | 159 | 1530 |  |

**Supplementary Table S2.** Confusion matrix for the predictions of the BiomedBERT model fine-tuned.

|  |  | **Actual class** | |  |
| --- | --- | --- | --- | --- |
|  |  | Positive | Negative |  |
| **Predicted class** | Positive | 131 | 23 | 154 |
|  | Negative | 28 | 1507 | 1535 |
|  |  | 159 | 1530 |  |

**Supplementary Table S3.** Confusion matrix for the predictions of the BiomedBERT-large model fine-tuned.

|  |  | **Actual class** | |  |
| --- | --- | --- | --- | --- |
|  |  | Positive | Negative |  |
| **Predicted class** | Positive | 135 | 35 | 170 |
|  | Negative | 24 | 1495 | 1519 |
|  |  | 159 | 1530 |  |

**Supplementary Table S4.** Confusion matrix for the predictions of the BioLinkBERT model fine-tuned.

|  |  | **Actual class** | |  |
| --- | --- | --- | --- | --- |
|  |  | Positive | Negative |  |
| **Predicted class** | Positive | 122 | 20 | 142 |
|  | Negative | 37 | 1510 | 1547 |
|  |  | 159 | 1530 |  |

**Supplementary Table S5.** Confusion matrix for the predictions of the BioM-BERT model fine-tuned.

| **Model name** | **F1-score** | **Precision** | **Recall** |
| --- | --- | --- | --- |
| **BiomedBERT** | 0.05 ± 0.08 | 0.11 ± 0.15 | 0.03 ± 0.06 |
| **BiomedBERT-large** | 0.15 ± 0.13 | 0.8 ± 0.40 | 0.09 ± 0.08 |
| **BioLinkBERT** | 0.20 ± 0.17 | 0.55 ± 0.45 | 0.12 ± 0.10 |
| **BioM-BERT** | 0.0 ± 0.0 | 0.0 ± 0.0 | 0.0 ± 0.0 |

**Supplementary Table S6.** Average performances of the models on the DUVEL test set with the data sets mimicking the distribution obtained with a random sampling of the annotation instead of with the active learning process (i.e., ~1% of positive instances over all the instances in the training set). Average and standard deviations were computed over 5 simulated data sets. Standard F1-score, precision and recall are computed on the test set after fine-tuning of the models. Results were rounded at the second decimal.

# References for the Supplementary Information

1.	[Herrero-Zazo, M., Segura-Bedmar, I., Martínez, P., et al. (2013) The DDI corpus: an annotated corpus with pharmacological substances and drug-drug interactions. *J. Biomed. Inform.*, **46**, 914–920.](http://paperpile.com/b/PAVGdB/4hZc)

2.	[Nachtegael, C., De Stefani, J. and Lenaerts, T. (2023) A study of deep active learning methods to reduce labelling efforts in biomedical relation extraction. *PLoS One*, **18**, e0292356.](http://paperpile.com/b/PAVGdB/ICbO)

3.	[Scheffer, T., Decomain, C. and Wrobel, S. (2001) Active hidden Markov models for information extraction. *Advances in Intelligent Data Analysis*, Lecture notes in computer science, Springer Berlin Heidelberg, Berlin, Heidelberg, pp. 309–318.](http://paperpile.com/b/PAVGdB/HadL)

4.	[Gu, Y., Tinn, R., Cheng, H., et al. (2022) Domain-specific language model pretraining for biomedical natural language processing. *ACM Trans. Comput. Healthc.*, **3**, 1–23.](http://paperpile.com/b/PAVGdB/aqUV)

5.	[Reyes, O., Altalhi, A.H. and Ventura, S. (2018) Statistical comparisons of active learning strategies over multiple datasets. *Knowl. Based Syst.*, **145**, 274–288.](http://paperpile.com/b/PAVGdB/FiSO)
